# Trends in Tick-Borne Virus Ecology: Vector Incrimination, Focal Distribution, and Reservoir Associations

**DOI:** 10.3390/v18080921

**Published:** 2026-08-21

**Authors:** Rachel E. Lange, Alan P. Dupuis, Alexander T. Ciota

**Affiliations:** 1 Arbovirus Laboratory, Wadsworth Center, New York State Department of Health, Slingerlands, NY 12159, USA; rachel.lange@health.ny.gov or rlange@albany.edu (R.E.L.); alexander.ciota@health.ny.gov (A.T.C.); 2 Department of Biomedical Sciences, College of Integrated Health Sciences, State University of New York University at Albany, Albany, NY 12222, USA

**Keywords:** tick-borne viruses, virus ecology, reservoir, tick-borne encephalitis virus, Crimean–Congo Hemorrhagic Fever virus, Severe Fever with Thrombocytopenia virus, Colorado Tick Fever virus, Powassan virus, Heartland virus, Bourbon virus

## Abstract

Ticks are prominent hosts for many viruses that are pathogenic to humans on almost every continent. Despite the documented historical association of ticks with viral disease in humans, clinical case reports are low, likely due to under-recognition of these pathogens. A key feature of understanding tick-borne virus transmission to humans lies in the natural invertebrate and vertebrate hosts supporting ecological maintenance. As humans are often infected as incidental hosts during the tick–virus life cycle, determining relevant ticks and mammals involved in maintenance can inform prevention strategies, surveillance priorities, and diagnostic assay development. This review aims to cover common trends in tick-borne virus ecology, including the wide breadth of implicated tick species, an unclear role for definitive vertebrate reservoirs, and the focal distributions of these viruses closely linked to these host ecological features. Here we also provide examples of prominent tick-borne viruses causing human disease between the Old (Africa, Asia, Europe) and New (Americas) Worlds, highlighting parallels in initial discoveries, vectors, and vertebrates including tick-borne encephalitis, Crimean–Congo hemorrhagic fever, severe fever with thrombocytopenia syndrome, Colorado Tick fever, and Powassan, Heartland, and Bourbon viruses.

## 1. Role of Ticks as Vectors for Viral Disease

The complicated role of arthropods in human disease transmission has been documented for centuries, from the recorded spread of fevers by biting flies in Nazareth during Biblical times to descriptions of fly blindness in ancient Egyptian texts [1,2]. The late 17th century saw great advances in this understanding as diseases like malaria (caused by *Plasmodium falciparum*) and filariasis (caused by *Wucheraria bancrofti*) were linked to mosquitoes biting humans [3]. One of these seminal discoveries was the described transmission of *Babesia bigemina*, the causative agent of Texas cattle fever, by *Boophilus annulatus*, now commonly referred to as the cattle fever tick [4]. The last 125 years since this report has been marked by an abundance of discoveries related to ticks and human pathogens ranging from early reports of bacterial and protozoan diseases like relapsing fever (*Borrelia hermsii*), East Coast fever (*Theileria parva*), and Rocky Mountain spotted fever (*Rickettsia rickettsii*) in *Ornithodoros*, *Rhipicephalus* and *Dermacentor* species to more recent discoveries like the association of Lyme disease (caused by *Borrelia burgdorferi* sensu lato) and human Ehrlichiosis (caused by *Ehrlichia* species) with *Ixodes scapularis* [5,6,7,8,9]. Initial reports of the association of ticks and viruses also occurred during this phase of vector-pathogen discovery. The identification of *Ixodes* species in the transmission of Russian Spring-Summer Encephalitis virus (now referred to as tick-borne encephalitis virus [TBEV]) across Russia and Central Europe marked the description of the second known arbovirus following yellow fever virus [10,11]. Soon after the discovery of the association of TBEV and ticks in the Old World, Colorado Tick Fever and Powassan viruses were discovered in geographically separate regions of North America [12,13,14]. Today, tick-borne viruses are reported from a wide range of tick genera on almost every continent except Antarctica [15,16]. The study of these interactions is unique compared to other common vector-borne pathogens transmitted by flying insects, as ticks are physiologically and ecologically distinct.

### 1.1. Tick Natural Histories

Arachnids diverged from insects over 500 million years ago, predating terrestrial evolution [17]. Their physiology is highly distinct from flying insects characterized by winged appendages, tri-segmented bodies, and 6 legs [18]. Arachnids are typically characterized by four pairs of legs (8), fewer body segments (2), and the absence of antennae and winged appendages; instead, they have specialized features like fangs and claws [19]. Within Arachnida, ticks (Order: Ixodida) are split into two main families, the hard (Ixodidae) and soft (Argasidae) ticks, differentiated by key morphological differences like the presence of a hard scutal plate (Ixodidae) and anatomical placement of the mouthparts [19]. Recently, two ancient families (Nuttalliellidae and Khimairidae) have also been described [20]. The natural history of Ixodids and Argasids involves three life stages (larvae, nymph, and adult) and the requirement of a bloodmeal from a vertebrate source to complete each developmental stage, reproduce, and lay eggs [21]. The full life cycle can take years, and ticks can survive for long periods without feeding or being associated with a vertebrate host [19,21]. Host-seeking and preference behaviors differ between families, genera, species, and even life stages. In relation to vertebrate hosts, ticks are often categorized in one-, two-, or three-host tick life cycles [19,21,22]. For example, in a one-host life cycle (i.e., *Rhipicephalus annulatus*), all life stages feed on a single host, while in a three-host life cycle (i.e., *Amblyomma variegatum*), each motile life stage will feed on a different host to complete stage development or egg production. Vertebrate hosts are fed upon unevenly, with different preferences for different life stages [22]. Ticks may be host-specific or opportunistic and further defined by feeding on one class, order, or even genus of hosts (i.e., *Ixodes marxi* preference for Sciuridae) or feeding on a wide spectrum of terrestrial hosts (i.e., *Ixodes ricinus*) [19,21,22]. These differences in host preference also translate to hunting behaviors with different strategies employed; for example, *Hyalomma* species use visual and chemical cues to actively follow and track new bloodmeal hosts while *Ornithodoros* species wait passively in host nests or burrows [21,23,24]. These host-feeding behaviors are also heavily influenced by other ecological factors like host abundance and diversity and environmental factors (i.e., habitat suitability for both vector and vertebrate) and seasonal changes that induce inactive periods for the tick called diapause. Tick behavior and the ecological relationship with their vertebrate hosts not only influence the life cycle but also impact the circulation and spread of viruses within hosts.

### 1.2. Virus Transmission in Ticks

Maintenance of a viral pathogen between ticks and their vertebrate hosts requires two transmission routes: (1) horizontal transmission, in which a tick can acquire or transmit a virus through feeding on a susceptible vertebrate host, and (2) vertical transmission, in which virus can pass in this tick through different life stages either through transovarial (mother to offspring) or transstadial (larvae to nymph, nymph to adult) [19,21,22,25]. Despite causing disease, humans are often involved in accidental or tangential transmission routes that involve a tick infecting a dead-end host [22]. In the case of tick-borne viruses, humans are considered dead-end hosts on the premise that they do not develop significant long-term viremia during infection or become infested with enough co-feeding ticks to facilitate transmission to naïve ticks. Therefore, to be an effective vertebrate reservoir, the host must be susceptible to infection, amplify and maintain the virus for a sufficient amount of time to infect naïve tick hosts, and exist in relative abundance and density sufficient to support relevant vector populations [19,21,22,25]. Many tick-borne viruses elicit rapid, transient viremia in wildlife, suggesting these vertebrates may not be reservoir competent despite being important hosts for the local tick population [22,26,27]. Non-viremic or reservoir-incompetent hosts have been shown to play an important role in an alternative mechanism of transmission, co-feeding transmission, where infected ticks feeding on a shared host can transmit virus proximally to uninfected, co-feeding ticks [28,29,30]. Tick–virus or vertebrate–virus interactions are further influenced by ecological and environmental factors that constrict tick activity and density and abundance of susceptible hosts [19,21,22,27].

## 2. Tick-Borne Viruses of the Old World

**Figure 1:**
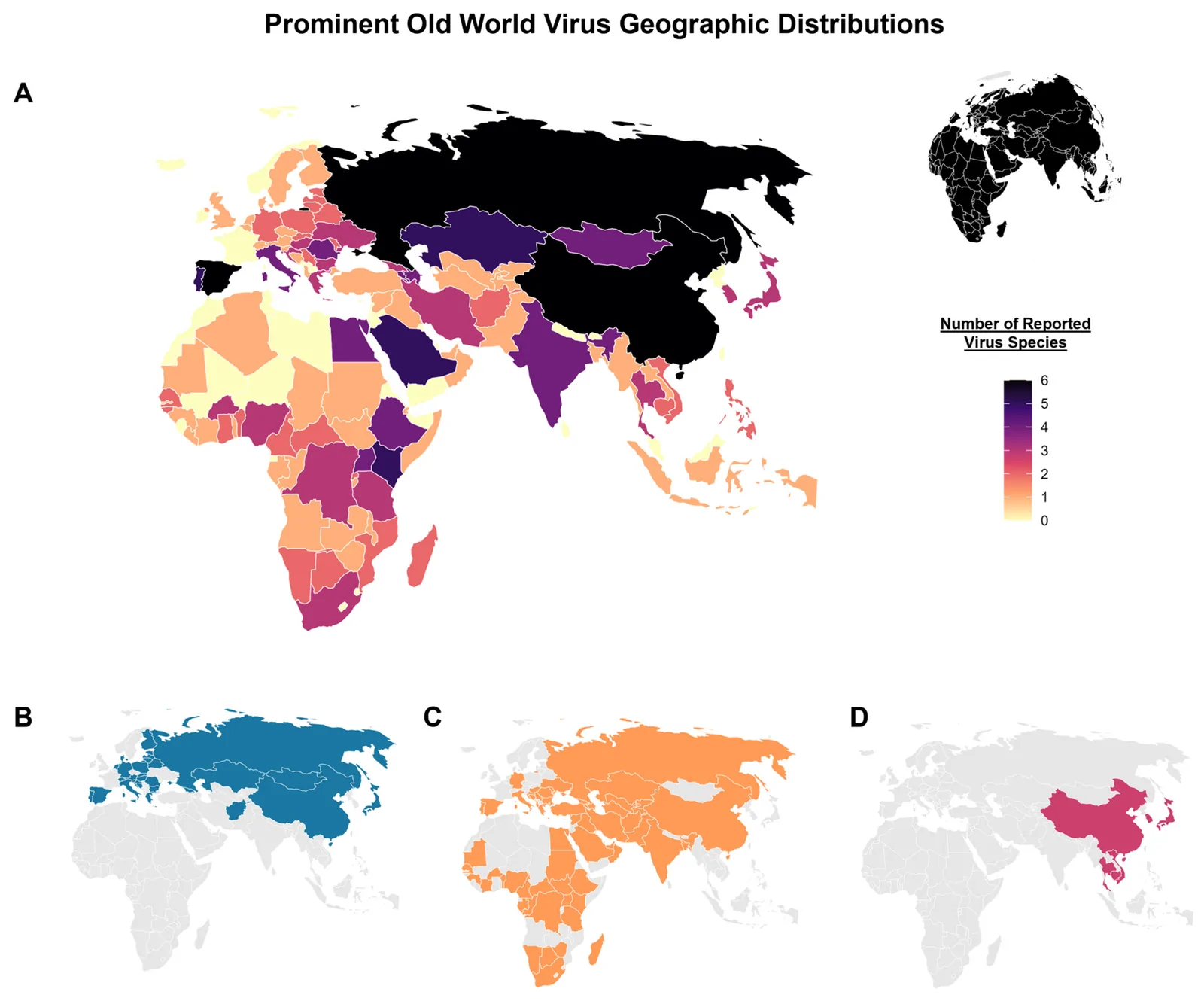
Geographic distribution of prominent tick-borne viruses circulating in the Old World, particularly on the African, Asian, and European continents. Range based on reported human cases, seropositive vertebrates, or virus-positive ticks at the country level. (**A**) Number of viruses currently reported in each country, ranging from 0 to 7 tick-borne viruses. (**B**) Geographic range of tick-borne encephalitis virus (TBEV, *Flaviviridae*, blue), (**C**) Crimean–Congo Hemorrhagic Fever virus (CCHFV, *Nairoviridae*, orange), and (**D**) Severe Fever with Thrombocytopenia Syndrome virus (SFTSV, *Phenuviridae*, pink).

A wide range of viruses are found circulating in ticks throughout the Old World, including Africa, Europe, and Asia (Figure 1, Table 1). Notably, viruses from the families *Flaviviridae*, *Phenuviridae*, *Nairoviridae*, and *Orthomyxoviridae* account for many of the viral diseases identified in humans or animal hosts (Table 1). The distribution and number of co-circulating viruses are based on reported cases and the presence of infected ticks or vertebrates, which likely underestimates the true distribution of these viruses and reported country-specific tick-borne virus burdens (Figure 1). Many factors influence this underestimate, including lack of reporting, testing, and awareness in some countries. Tick-borne virus disease presentation is often associated with either neurological sequelae or hemorrhagic fevers, but reports of severe cases are low, with only three viruses accounting for over 1,000 annual human case reports (TBEV, Crimean–Congo Hemorrhagic Fever virus, and Severe Fever with Thrombocytopenia Syndrome virus). As is common with arboviruses, this is presumably an underestimate, as most human infections are likely asymptomatic, self-resolving, or undiagnosed. Additionally, there are numerous known tick-borne viruses in the Old World with probable pathogenic potential in humans, like Eyach, Tribec, Karshi, Batken, and Kadam viruses, but lack any or have minimal reports of confirmed clinical human cases, likely due to under-recognition and diagnostic biases. Table 1 highlights the broad geographic range of these viruses and the diverse range of tick species known to transmit or harbor each virus based on field and experimental studies. These include hard and soft ticks from distinct genera that display different feeding behaviors, hunting strategies, and ecological preferences. Little is known about the reservoirs of these viruses. Most host associations are linked to disease, as seen with African Swine Fever and Nairobi Sheep Disease viruses in suids (i.e., wild boars and domesticated pigs) and ungulates (i.e., sheep and goats), respectively.

### 2.1. Tick-Borne Encephalitis Virus

The discovery of ticks associated with the transmission of an encephalitic virus in 1937 marked the first recognized viral infection in humans transmitted by ticks [Spring-Summer Epidemic Encephalitis (*Flaviviridae*)] [10]. As such, much of our understanding about tick-borne virus ecology and transmission is based on the study of this virus now referred to as TBEV. Clinical accounts of encephalitic diseases of unknown etiology were reported across Russia and Central Europe between the 1890s–1920s [31,32,33]. It was not until the 1940s that this disease was understood to be caused by a virus associated with the tick vectors *Ixodes persulcatus* and *I. ricinus*, supported by the observation of outbreak occurrence in the summer, high disease prevalence among outdoor forest workers, and field studies incriminating these species in Russia [10,11,34]. Further investigation into the enzootic cycle of ticks and TBEV revealed novel hypotheses for tick-borne pathogen transmission, including the role of co-feeding transmission on non-viremic mammalian hosts and focal disease maintenance [35,36]. To date, TBEV comprises three major subtypes (European, Far Eastern, and Siberian) with multiple additional subtypes proposed recently [37]. These subtypes present a wide range of symptoms in humans, with the Far Eastern subtype associated with severe neurological sequelae and the European subtype primarily causing asymptomatic or self-resolving acute febrile illness [38]. The divergence of these subtypes is proposed to be linked to vector switching between different *Ixodes* species (*persulcatus* and *ricinus*) and maintenance in geographically isolated foci (Central Europe and Siberian Russia) [39]. Another important aspect of TBEV ecology is the unknown contributions of a mammalian reservoir. Current reports suggest small rodent species (*Apodemus*, *Myodes*, and *Sorex*) involvement [40,41,42]. Definitive evidence for any of these hosts is lacking but highlights a potential maintenance strategy that involves ticks, small mammals, and densely forested regions. Lastly, as depicted in Table 1, additional tick species have been associated with TBEV, including *Haemaphysalis concinna* and *Dermacentor reticulatus* [43,44]. TBEV illustrates the focal nature, diverse vector competence, and elusive mammalian involvement often associated with tick-borne viruses circulating in the Holarctic region.

### 2.2. Crimean–Congo Hemorrhagic Fever Virus

Following the identification of TBEV, another disease of unknown etiology was identified in the 1940s in Soviet soldiers re-occupying regions of the Crimean Peninsula [45]. Similar reports continued throughout Russia and Bulgaria in the 1940–60s until the agent was isolated, characterized, and determined to be a bunyavirus identical to a virus isolated in the Democratic Republic of the Congo in 1956, Congo virus [46,47,48]. The virus was re-named Crimean–Congo Hemorrhagic Fever virus (CCHFV, *Nairoviridae*), now recognized as the most widespread and genetically diverse tick-borne virus circulating globally [49,50,51]. Clinical disease is associated with hemorrhagic fever symptoms, which in severe cases include vascular leakage, multi-organ failure, and shock [49]. Transmission is associated with tick bites or direct contact with infected fluids (i.e., animal blood). Human cases have been reported across Central Asia, Southern Europe, the Balkans, the Middle East, and Central Africa. Of note, divergent genetic lineages are linked with spatially distinct endemic regions separated by prominent geographic features like oceans or mountain ranges [49]. Widespread endemicity is associated with the distribution of the *Hyalomma* tick species complex (i.e., *Hyalomma marginatum* in Eurasia and *Hyalomma marginatum rufipes* in Africa). *Hy. marginatum* is known to tolerate diverse environments including savannas and deserts, aggressively bites humans, and feeds on a variety of small and large mammals [49,52]. Other ticks have been implicated in enzootic maintenance, including *Ixodes*, *Dermacentor*, and *Rhipicephalus* species, with a particular focus in Moldova highlighting an atypical enzootic focus in a deciduous forested region [52]. Experimental evidence supports CCHFV maintenance in these ticks through transstadial and transovarial transmission, with vertebrate hosts hypothesized to play a minimal role in maintenance and evolution [49]. Serological studies have revealed that livestock are sensitive sentinels for CCHFV circulation and risk, as they are heavily parasitized by *Hyalomma* ticks and create a bridge between infected ticks and humans through agricultural practices. Experimental infections indicate short-term viremia and lack of clinical illness in mammals such as sheep, hares, and calves, further suggesting virus maintenance and transmission to humans is primarily driven by the tick host [49,52,53]. More importantly, the evidence of long-distance viral migration between geographically isolated foci may be linked to the international transport of agricultural hosts, which has significantly increased during the 21st century [49]. While TBEV represents a tick-borne virus maintained in forested regions with tick-small mammal transmission cycles, CCHFV is maintained in an alternative ecological niche defined by arid savanna/desert regions and a tick-ungulate transmission cycle. Despite this ecological distinction, common themes emerge for CCHFV, including broad vector competence, potential limited mammal involvement in virus amplification, and genetically distinct focal maintenance.

### 2.3. Severe Fever with Thrombocytopenia Syndrome Virus

In recent decades, another novel bunyavirus was identified circulating throughout East Asia, Severe Fever with Thrombocytopenia Syndrome virus [SFTSV, *Phenuviridae*, formerly Huaiyangshan virus and now recognized as Dabie bandavirus by ICTV]. An outbreak of patients with thrombocytopenia and leukopenia in China in 2009 of unknown etiology was later determined to be a novel bunyavirus circulating in *Haemaphysalis longicornis* [54,55]. While *Ha. longicornis* remains the primary incriminated vector, SFTSV has been detected in *R. microplus, A. testudinarium*, and *I. nipponensis* [56,57]. Outside of China, SFTSV has been reported in Vietnam, Thailand, Cambodia, South Korea, and Japan either through virus-positive ticks, mammals, or clinical cases [58,59,60]. Similar to TBEV, the isolated geographic foci of SFTV have been associated with genetically distinct genotypes (7 to date) that differ in disease severity (i.e., case fatality rates for SFTSV China: 8% vs. Japan: 27%) [61,62,63,64]. Migratory birds in the East Asian-Australasian flyway have been implicated in dispersal of ticks and subsequently SFTSV [65,66]. With such geographically isolated and disparate regions of reported SFTSV circulation, a diverse range of mammalian hosts have been implicated as potential reservoirs or amplifying hosts, including ruminants, rodents, domestic pets, and hedgehogs [56,67,68,69,70]. Field and experimental studies support a prominent role of agricultural livestock, particularly goats, in SFTSV maintenance in China. Current hypotheses suggest genetic diversity due to recombination events may occur in these hosts [48,70,71]. Studies with SFTSV further highlight important concepts in tick-borne virus ecology, including the role of migratory birds in tick and pathogen dispersal, a diverse range of competent tick and mammalian hosts, and differences in clinical disease associated with geographically isolated genotypes.

## 3. Tick-Borne Viruses of the New World

**Figure 2:**
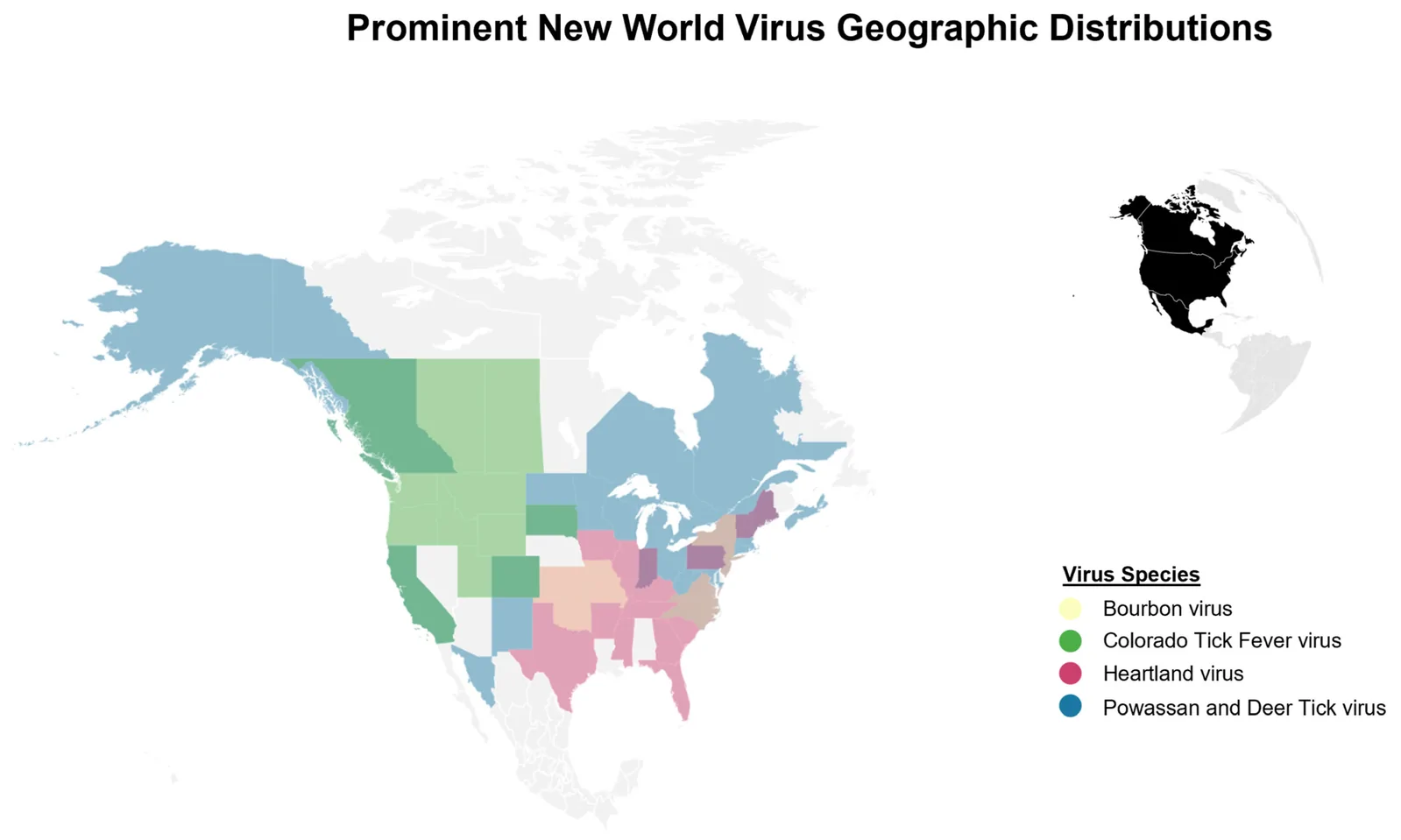
Geographic distribution of prominent tick-borne viruses circulating in the New World, particularly North America. Range is based on reported human cases, seropositive vertebrates, or virus-positive ticks at the state (United States of America, Mexico) or province (Canada) level. The four circulating viruses include Bourbon virus (BRBV, *Orthomyxoviridae*, yellow), Colorado Tick Fever virus (CFTV, *Spinareoviridae*, green), Heartland virus (HRTV, *Phenuviridae*, pink), and Powassan and Deer Tick viruses (POWV/DTV, *Flaviviridae*, blue).

There are only four tick-borne viruses known to be pathogenic to humans circulating in the New World, particularly in North America: Colorado Tick Fever virus (CTFV, *Spinareoviridae*), Powassan virus (POWV or DTV, *Flaviviridae*), Heartland virus (HRTV, *Phenuviridae*), and Bourbon virus (BRBV, *Orthomyxoviridae*) (Figure 2, Table 2). Despite the abundant circulation of mosquito-borne viruses in Central and South America, little remains known about the burden of tick-borne viruses in these regions, despite the known prevalence of *Amblyomma*, *Rhipicephalus*, and *Dermacentor* species. The current knowledge of pathogenic tick-borne viruses in the Americas is dependent on knowledge of the four circulating viruses in the North American continent, likely due to recognition of fatal or severe clinical cases, support from robust awareness and testing for bacterial tick-borne pathogens, and lack of prioritization of ticks in the mosquito-dominated tropics.

### 3.1. Colorado Tick Fever Virus

Colorado Tick Fever virus (CTFV) was the first recognized and described North American tick-borne virus of the Coltivirus genus [72]. The initial detection occurred in 1943 from the blood of a patient from Colorado [72,73]. Colorado Tick Fever is a biphasic self-limiting febrile illness in humans that can progress to lethal hematological and central nervous system abnormalities [74,75]. Fewer than 15 human cases in the US are reported each year, and 6 states account for 80% of cases (Wyoming, Montana, Utah, Oregon, Colorado, and Idaho) [76]. The human epidemiology of CTFV overlaps with the geographic range of the primary vector, the Rocky Mountain wood tick (*D. andersoni*), which is established across the Rocky Mountains region of the USA and southern portions of British Columbia, Alberta, and Saskatchewan in Canada [74]. *D. andersoni* was implicated as the primary vector in Colorado in the 1950s, with CTFV infection rates (5–40%) remaining stable over time in endemic states [12,77,78,79,80]. Additional studies also identified CTFV in other *Dermacentor* and *Ixodes* species from the western USA, but little is known about their role in viral maintenance or transmission [81,82,83]. The primary vertebrate host involved in CFTV is the golden-mantled ground squirrel (*Callospermophilus lateralis*). Notably, experimentally infected ground squirrels were shown to maintain long-term viremia (<100 days) with no clinical signs under overwintering/hibernation conditions, suggesting a viable role for these vertebrate species in maintenance and transmission to tick hosts [84]. Field and experimental studies have implicated additional vertebrate hosts, including chipmunk, woodrat, hare, vole, and other ground squirrel species, though their role in CTFV transmission and maintenance is unknown [82].

### 3.2. Powassan and Deer Tick Virus

In 1958, ten years after the discovery of CTFV in Colorado, Powassan virus (POWV) was isolated from the brain of a fatal pediatric case from the North Bay region of Ontario, Canada [85]. Human disease resulting from POWV primarily affects the central nervous system, but most cases are presumed to be asymptomatic or self-resolving in the acute stage [86]. To date, human cases have only been reported in the Northeast and Midwest regions of the USA and southeastern portions of Canada, with the majority of cases reported from Massachusetts, New York, Minnesota, and Wisconsin [87]. Similar to CTFV, annual reported human cases of POWV are low but have increased since 2000 [86,88,89,90]. The increase in cases is hypothesized to be attributed to changing vector dynamics. POWV was historically reported only in nidicolous Ixodid ticks (*I. marxi* and *I. cookei*), which rarely encounter humans, but range expansion of the aggressive human-biting *I. scapularis* throughout the Northeast and Midwest in the late 1900s has now highlighted this tick as a primary vector in terms of transmission to humans [91]. Additionally, the identification of POWV in *I. scapularis* revealed the existence of two distinct lineages, now termed POWV lineage 1 and deer tick virus (DTV) or POWV lineage 2 [92]. The current focus of POWV surveillance and control is *I. scapularis*, though both POWV and DTV have been isolated from other tick species including *Dermacentor* and *Haemaphysalis* species [93,94,95]. The role of a vertebrate reservoir, however, remains unknown, with a broad range of forest fauna and mesomammals reported to be seropositive for POWV or harbor ticks positive for POWV [96,97].

### 3.3. Heartland and Bourbon Viruses

Recently, two other pathogenic tick-borne viruses have been recognized in North America, both from the Midwestern USA and associated with the Lone Star tick, *Amblyomma americanum:* Heartland (*Phenuiviridae*) and Bourbon (*Orthomyxoviridae*) viruses [98]. Heartland and Bourbon viruses (HRTV and BRBV) were first identified in 2009 in Missouri and 2014 in Kansas, respectively, in human patients presenting with febrile illness and hematological abnormalities [99,100]. To date, human cases of HRTV have been reported in 14 states spanning the geographic range of *A. americanum* [98,101], and BRBV infections have been reported in just three states in the Midwest [100,102,103]. Infections with both viruses have subsequently been identified through retrospective testing outside of the Midwest in New York State [104]. Case reports likely underestimate the true range of both HRTV and BRBV, as virus-positive *A. americanum* have been detected beyond these known foci in Georgia, New York, New Jersey, Illinois, Alabama, and Virginia [105,106,107,108,109]. With no other tick species implicated in HRTV or BRBV transmission (except one report of spillover of BRBV into *H. longicornis* in Virginia), the geographic range of *A. americanum* is likely a good reflection of where human risk occurs [108,110]. Additionally, in the absence of any known vertebrate involvement, white-tailed deer (*Odocoileus virginianus*) have acted as key sentinel hosts for identification of HRTV/BRBV circulation in combination with tick surveillance efforts [98,111,112].

## 4. Tick-Borne Viruses of Unknown Pathogenic Potential in the New World

While only four tick-borne viruses are known to cause disease in humans in North America, a diverse range of other viruses have been identified in hard and soft ticks, including members of the *Phenuviridae*, *Nairoviridae*, *Rhabdoviridae*, *Sedoreoviridae* and *Orthomyxoviridae* families (Table 3). These viruses are often harbored by ticks that almost exclusively feed on one host, such as *Haemaphysalis leporispalustris* or *Ixodes dentatus* and lagomorphs (i.e., Silverwater virus, Connecticut virus, New Minto virus), or *Ixodes uriae* and seabirds (i.e., Great Island virus, Bauline virus, Hughes virus) [13,113,114,115,116]. The discovery of these viruses is often a result of mammal surveys conducted in the heavily forested eastern portions of the USA and Canada or field investigations in seabird colonies along the Pacific coastline. For example, the discovery and identification of Silverwater virus was a result of virus isolation from snowshoe hares and *Haemaphysalis leporispalustris* collected during field investigations into the ecology of Powassan virus [13]. Viruses vectored by soft ticks are even more elusive, as these ticks feed for shorter periods on vertebrate hosts and usually reside in burrows or nests, limiting their exposure to human activities [117]. For example, in Texas, *Argas cooleyi* collected from swallow nests resulted in the discovery of Sixgun City virus and Aransas Bay virus was discovered in *Ornithodoros capensis* collected from coastal bird nests [118,119]. As many of these ticks do not often encounter or feed on humans, their pathogenicity remains unknown. Recent work, however, has identified potential roles for these viruses in human disease, such as recent associations of fatal encephalitic cases with Lone Star virus infections [120]. These examples highlight the diversity of tick-borne viruses circulating throughout North America and how tick behavior can limit human exposure, despite these viruses potentially being pathogenic.

## 5. Conclusions and Future Priorities

There is growing public health concern related to tick-borne viruses that is not isolated to any particular geographic region, tick species, or reservoir. While this review covers the current vector associations, distributions, and vertebrate host involvement for tick-borne viruses known to cause human disease burdens, many important gaps remain to further understand the ecological dynamics of these viruses. With a broad range of tick genera and species associated with these pathogens, the significance of each species’ role in transmission to humans and within natural cycles is important for defining species of concern. Additionally, host–virus dynamics are also poorly resolved, and there is evidence that different tick species drive the evolution of particular viruses like TBEV, yet little is known on a broader scale about species-specific evolutionary pressures. Not only can these interactions drive viral evolution in the tick host, but these changes can also alter pathogenicity in humans. The geographically focal distributions of tick-borne viruses also reflect the important role of tick ecology on enzootic maintenance and ultimately disease transmission; yet human diagnostics and comprehensive surveillance to accurately define transmission hotspots are sparse and often only evaluated after fatal human cases. Much also remains to be understood about vertebrate involvement in tick-borne virus transmission and circulation, as potential reservoir associations to date are often based on serological results or viral isolations from a small number of hosts. Serological positivity and/or evidence of viral infection in nature does not constitute true reservoir or amplifying host status and experimental infections are limited, as relevant tick-borne virus hosts of interest are wildlife species with no model organism counterparts. With multiple novel tick-borne viruses reported almost every year, it is important to prioritize these questions to further elucidate these complex ecologies and prevent continued expansion of tick-borne viral disease in humans.

## Figures and Tables

**Table 1:** Prominent tick-borne viruses of the Old World. List of tick-borne viruses known to infect humans and/or animals in the Old World, including Africa, Europe, and Asia. All associated hosts and case burdens are based on reports of these viruses as of 2025.

Virus *Family*	Geographic Distribution	Vector Species (Primary)	Potential Reservoir or Intermediate Hosts
African Swine Fever Virus (*Asfarviridae*)	Africa Eurasia Southeast Asia	*Ornithodoros* species (*moubata, erraticus, parkeri*)	Suids
Alkhumra Hemorrhagic Fever Virus (*Flaviviridae*)	Africa Middle East	*Amblyomma lepidum* *Hyalomma* species (*dromedarii, rufipes*) *Ornithodoros savignyi*	Camelids
Bhanja Virus (*Phenuviridae*)	Africa Asia Europe	*Amblyomma variegatum* *Dermacentor marginatus* *Haemaphysalis* species *Hyalomma* species *Rhipicephalus* species	Ruminants Hedgehogs Squirrels
Crimean–Congo Hemorrhagic Fever Virus (*Nairoviridae*)	Africa Asia Europe (East) Middle East	*Amblyomma variegatum* *Dermacentor* species *Haemaphysalis* species *Hyalomma* species *Ixodes* species *Ornithodoros* species *Rhipicephalus* species	Ruminants
Dhori Virus (*Orthomyxoviridae*)	Asia Europe (South) Middle East	*Dermacentor marginatus* *Hyalomma* species (*dromedarii, marginatum,* *schulzei*, etc.)	Camelids
Kemerovo Virus (*Reoviridae*)	Asia Europe (East)	*Dermacentor reticulatus* *Ixodes* species (*persulcatus, ricinus*, etc.)	Unknown
Kyasanur Forest Disease Virus (*Flaviviridae*)	Asia	*Dermacentor auratus* *Haemaphysalis* species (*kyasanurensis, spinigera,* *wellingtoni,* etc.) *Hyalomma marginatum* *Ixodes petauristae* *Rhipicephalus haemaphysaloides*	Rodents Primates
Langat Virus (*Flaviviridae*)	Asia	*Ixodes granulatus* *Haemaphysalis* species (*papuana, longicornus*)	Unknown
Louping Ill Virus (*Flaviviridae*)	Europe (West) Japan Russia	*Ixodes* species (*ricinus, persulcatus*, etc.) *Rhipicephalus appendiculatus*	Ruminants Lagomorphs Rodents
Nairobi Sheep Disease Virus [Dugbe/Ganjam Virus] (*Nairoviridae*)	Africa (Central) Asia	*Amblyomma* species (*variegatum, gemma, lepidum*) *Haemaphysalis* species (*intermedia, longicornis, wellingtoni*) Rhipicephalus species (*appendiculatis, pulchellus,* *haemaphysaloides*, etc.)	Ruminants
Omsk Hemorrhagic Fever Virus (*Flaviviridae*)	Asia (primarily Russia)	*Dermacentor* *Ixodes*	Rodents
Dabie bandavirus [Severe Fever with Thrombocytopenia Syndrome Virus] (*Phenuviridae*)	Asia (East)	*Amblyomma testudinarium* *Haemaphysalis* species (*concinna, longicornus,* *hystricis*, etc.) *Ixodes nipponensis* *Rhipicephalus microplus*	Ruminants Hedgehogs
Tamdy Virus (*Nairoviridae*)	Asia	*Haemaphysalis concinna* *Hyalomma* species (*asisticum, anatolicum,* *marginatum*, etc.) *Rhipicephalus turanicus*	Camelids
Tick-borne Encephalitis Virus (*Flaviviridae*)	Asia Europe Middle East	*Dermacentor* species (*reticulatus, marginatus,* *silvarum,* etc.) *Haemaphysalis* species (*concinna, longicornis,* *punctata*, etc.) *Ixodes* species (*ricinus, persulcatus,* *ovatus,* etc.)	Rodents
Thogoto Virus (*Orthomyxoviridae*)	Africa Europe (South)	*Amblyomma variegatum* *Haemaphysalis* species (*longicornis, spinulosa*) *Rhipicephalus* species (*appendiculatus, bursa,* *sanguineus*, etc.)	Unknown

**Table 2:** Prominent tick-borne viruses of the New World. List of tick-borne viruses known to infect humans and/or animals in the New World, including North and South America. All associated hosts and case burdens are based on reports of these viruses as of 2025.

Virus (*Family*)	Geographic Distribution	Vector Species (Primary)	Potential Reservoir Hosts
Colorado Tick Fever Virus (*Spinareoviridae*)	USA (West) Canada (Southwest)	*Dermacentor andersoni*	Golden-mantled ground squirrel
Powassan and Deer Tick Viruses (*Flaviviridae*)	USA (Northeast, Midwest) Canada (Southeast) Russia (Far East)	*Ixodes* species (*cookei, marxi, scapularis*); *Dermacentor* species (*andersoni, variabilis*)	Squirrels, shrews, various mustelids
Heartland Virus (*Phenuviridae*)	USA (Northeast, Central)	*Amblyomma americanum*	Unknown
Bourbon Virus (*Nairoviridae*)	USA (Northeast, Central)	*Amblyomma americanum*	Unknown

**Table 3:** Tick-borne viruses of the New World with unknown pathogenic potential in humans. List of select tick-borne viruses present in parts of the New World, specifically North America. The capacity for human disease remains unknown.

Virus (*Family*)	Geographic Distribution	Vector Species (Primary)	Potential Reservoir Hosts
Silverwater Virus (*Phenuiviridae*)	USA (Northeast) Canada	*Haemaphysalis leporis-palustris*	Lagomorphs (Snowshoe Hares)
Lone Star Virus (*Phenuiviridae*)	USA	*Amblyomma americanum*	Unknown
Connecticut Virus (*Rhabdoviridae*)	USA (Northeast)	*Ixodes dentatus*	Unknown (Potentially Lagomorphs—i.e., eastern cottontails)
Long Island tick rhabdovirus (*Rhabdoviridae*)	USA (Northeast)	*Amblyomma americanum*	Unknown
Great Island Virus (*Sedoreoviridae*)	Canada (Northeast)	*Ixodes uriae*	Unknown (Potentially seabirds—i.e., puffins)
Bauline Virus (*Sedoreoviridae*)	Canada (Northeast)	*Ixodes uriae*	Unknown (Potentially seabirds—i.e., puffins)
Sawgrass Virus (*Rhabdoviridae*)	USA (Southeast)	*Dermacentor variabilis* *Haemaphysalis leporis-palustris*	Unknown
Hughes Virus (*Nairoviridae*)	USA Caribbean Islands	*Ornithodoros* species (*capensis, denmarki*) *Ixodes uriae*	Seabirds (terns)
Estero Real Virus (*Nairoviridae*)	Cuba	*Ornithodoros tadaridae*	Unknown (Potentially bats)
Sixgun City Virus (*Sedoreoviridae*)	USA (Southwest)	*Argas cooleyi*	Unknown (Potentially birds—i.e., swallows)
Mono Lake Virus (*Sedoreoviridae*)	USA (West)	*Argas cooleyi*	Unknown (Potentially birds—i.e., swallows, gulls)
Sierra Nevada Virus (*Rhabdoviridae*)	USA (West)	*Ornithodoros coriaeceus*	Unknown
Pacific Coast Tick Virus (*Nairoviridae*)	USA (West)	*Dermacentor occidentalis*	Unknown
Farallon Virus (*Nairoviridae*)	USA (West)	*Ornithodoros* species (*capensis, denmarki*)	Unknown (Potentially seabirds—i.e., gulls)
New Minto Virus (*Rhabdoviridae*)	USA (Northwest)	*Haemaphysalis leporis-palustris*	Lagomorphs (Snowshoe hares)
South Bay Virus (*Nairoviridae*)	USA	*Ixodes scapularis*	Unknown
Aransas Bay Virus (*Orthomyxoviridae*)	USA	*Ornithodoros capensis*	Unknown

## Data Availability

All data is contained within the article. Code used to generate Figure 1 and Figure 2 in RStudio v.2025.05.1-513 are available upon request.

## Abbreviations

The following abbreviations are used in this manuscript:

BRBV	Bourbon virus
CCHFV	Crimean–Congo Hemorrhagic Fever virus
CTFV	Colorado Tick Fever virus
DTV	Deer Tick virus
HRTV	Heartland virus
POWV	Powassan virus
SFTSV	Severe Fever with Thrombocytopenia Syndrome virus
TBEV	Tick-borne Encephalitis virus
